# Detection of *Schistosoma japonicum* and *Oncomelania hupensis quadrasi* environmental DNA and its potential utility to schistosomiasis japonica surveillance in the Philippines

**DOI:** 10.1371/journal.pone.0224617

**Published:** 2019-11-20

**Authors:** Raffy Jay C. Fornillos, Marcello Otake Sato, Ian Kim B. Tabios, Megumi Sato, Lydia R. Leonardo, Yuichi Chigusa, Toshifumi Minamoto, Mihoko Kikuchi, Emelda R. Legaspi, Ian Kendrich C. Fontanilla

**Affiliations:** 1 DNA Barcoding Laboratory, Institute of Biology, College of Science, University of the Philippines Diliman, Quezon City, Philippines; 2 Natural Sciences Research Institute, University of the Philippines Diliman, P. Velasquez St. Diliman, Quezon City, Philippines; 3 Department of Tropical Medicine and Parasitology, Dokkyo Medical University, Tochigi, Japan; 4 College of Medicine, University of the Philippines Manila, Ermita Manilla, Philippines; 5 Graduate School of Health Sciences, Niigata, Japan; 6 Graduate School, University of the East Ramon Magsaysay Memorial Medical Center, Quezon City, Philippines; 7 Graduate School of Human Development and Environment, Kobe University, Tsurukabuto, Nada-ku, Kobe, Japan; 8 Department of Immunogenetics, Institute of Tropical Medicine, Nagasaki University, Sakamoto, Nagasaki, Japan; 9 Medical Zoology Laboratory, Schistosomiasis Research and Training Center, Palo Leyte, Philippines; University of Agricultural Sciences and Veterinary Medicine Cluj-Napoca, Life Science Institute, ROMANIA

## Abstract

In recent years, the prevalence and infection intensity of *Schistosoma japonicum* in endemic areas of the Philippines have significantly decreased due to yearly population-based treatment strategies, yet transmission rates remain high and uninterrupted. An important indicator of active disease transmission is the presence of *Schistosoma japonicum* and its snail intermediate host *Oncomelania hupensis quadrasi* in freshwater habitats. In this study, we sought to apply a species-specific real-time PCR (qPCR) assay for the detection of *S*. *japonicum* and *O*. *hupensis quadrasi* in freshwater samples using environmental DNA approach that can complement the commonly utilized malacological survey in determining potential transmission foci in order to have a more effective snail surveillance strategy for schistosomiasis japonica in endemic areas. The newly developed assay was specific to *S*. *japonicum* and *O*. *hupensis quadrasi* with no amplification detected against non-target trematode *Fasciola* spp. and snails such as *Lymnaea* spp., *Pomacea canaliculata*, and *Melanoides* spp. that typically co-exist in the same environment. The assay effectiveness was determined using 19 environmental water samples collected from Northern Samar (N = 5 sites), Leyte (N = 11 sites) and Compostela Valley (N = 3 sites) and compared to malacological survey for determining *O*. *hupensis quadrasi* snail colonies and snail crushing to visualize *S*. *japonicum* cercariae. TaqMan qPCR targeting a short fragment of the cytochrome *c* oxidase subunit 1 (*cox1*) gene was positive for *S*. *japonicum* in 9 sites, for *O*. *hupensis quadrasi* in 9 sites, and for both *S*. *japonicum* and *O*. *hupensis quadrasi* in 5 sampling sites. Moreover, it was able to detect *O*. *hupensis quadrasi* in 3 out of 12 sites found negative and 6 out of 7 sites found positive through malacological survey, and in 4 of the 5 snail sites positive for snails with cercariae. Overall, this method can complement malacological surveys for monitoring of schistosomes in endemic areas of the Philippines, especially those with high risk of human infection.

## Introduction

Schistosomiasis japonica remains to be a significant public health threat in the Philippines, causing morbidities in the majority of endemic rural communities and usually affecting school-children, farmers and fishermen [1–3]. Despite the national schistosomiasis prevalence in the Philippines is less than 1% the variation very high, with some areas in Northern Samar reporting 48% prevalence of the disease [2]. This parasitic infection is considered both a water-borne and a snail-borne zoonosis [2–4]. In the Philippines, the pomatiopsid snail *Oncomelania hupensis quadrasi* (Mollendörff, 1895) serves as the intermediate host of *Schistosoma japonicum* [5]. These snails can be found in both natural and man-made waterlogged and shady areas characterized by lush vegetation [1,6].

Presence of the snail intermediate host in water sources with human use implies potential transmission of the disease to humans since the infective larval stage, called cercaria, develops inside the snail and is shed into the surrounding freshwater Schistosomiasis japonica is considered an occupational risk whom freshwater fishermen and farmers are at high risk of getting the infection due to frequent contact with water associated in their work. Children are also at high risk due to frequent playing and water contact which exposes them to cercariae [2,6].

Mass drug administration of praziquantel in endemic areas remains a cornerstone strategy implemented by the Philippine Department of Health for controlling morbidities and mortality in human infections [7,8]. Other key strategies such as snail control through physical modification of the habitat and mollusciciding were proven effective but are very costly. Improving environmental sanitation by building facilities such as toilets and latrines and the provision of safe sources of water for domestic use can greatly reduce environmental contamination and exposure among high risk communities. Treatment of animals serving as reservoir hosts following a One Health approach is highly recommended to interrupt transmission of *S*. *japonicum* [9]. Lastly, campaigns for health promotion and awareness of the disease are also needed for a sustainable control program for schistosomiasis japonica [1–3]

The use of freshwater for the detection of both *S*. *japonicum* and *O*. *hupensis quadrasi* is a potential area to be explored since it serves as a medium used by *S*. *japonicum*’s miracidia and cercariae in locating suitable hosts for transmission to take place [1–3,10,11]. This strict requirement for water explains the focal distribution in areas with pronounced wet season with significant amount of rainfall throughout the year [8]. Thus, in the Philippines, schistosomiasis japonica is mostly endemic in provinces located in the southeastern part of the country, although new foci have been recently discovered in two provinces with pronounced dry season, providing possible evidence for adaptation of the snail intermediate host to this condition [12,13]. With malacological surveys currently serving as the conventional method to determine snail colonies and their infection rates, correct identification of *O*. *hupensis quadrasi* is paramount to ensure accuracy and reliability of data. There is also a need to complement the conventional method of searching for snails in known and potential snail sites into one that does not rely on skill in collection and identification as well as physical work [3,7].

Detection of cercarial DNA of *S*. *japonicum* from water samples was first reported as a promising technique to apply in schistosomiasis-endemic areas in China [14–16]; however, no trials or field tests have yet been done in the Philippines. Ecological surveys using environmental DNA (eDNA) demonstrates the presence and distribution of micro- and macroorganisms from environmental material such as water and soil samples [17–19]. Several studies have been conducted to optimize DNA detection of *S*. *japonicum* in both laboratory and field water samples utilizing various genetic markers. Utilization of a 176 base pair (bp) target sequence from a 3.9 kilobase (kb) long non-terminal retrotransposon of *S*. *japonicum* to amplify a defined number of cercariae isolated from laboratory-infected *O*. *h*. *chui* and *O*. *h*. *hupensis* was done in a novel PCR assay [14]. Other potential markers used for *S*. *japonicum* cercariae include non-terminal retrotransposons such as Merlin DNA transposon and a putative deoxyribodipyrimidine photo-lyase in conjunction with real-time PCR (qPCR) through TaqMan technology. Positive linear correlation was shown when field water samples collected from a non-endemic area and spiked with a known number of *S*. *japonicum* cercariae were compared with the qPCR detection method [15]. Modified field water sampling techniques using a floating direct sampler and a siphon sampler that could concentrate cercariae present in surface water were also performed [15]. Moreover, eDNA detection of *S*. *mansoni* from water samples collected in Ampisavankaratra, Madagascar was successfully carried out using a 162 bp fragment of the *cox1* gene [20]. In the same area where *S*. *mansoni* eDNA was detected, the presence of the snail intermediate host *Biomphalaria pfeifferi* was detected by malacological survey. Application of eDNA in field surveys was done not only in schistosomes but also in other trematode species such as *Opisthorchis viverrini* in tributaries along the Mekong River in Lao PDR [21] and *Fasciola hepatica* in some Welsh farms in the United Kingdom [22].

The use of eDNA from water samples could therefore be a safer and more reliable alternative in field surveys for schistosomiasis. This can minimize the potential risk associated with exposure to cercariae-contaminated waters during malacological surveys. With the use of eDNA detection from freshwater samples, this method can be optimized and used effectively in monitoring areas with significant transmission and near elimination status of the disease, which sometimes cannot be addressed through conventional methods [15,20,23]. This study therefore aimed to develop an eDNA detection system for *S*. *japonicum* and *O*. *hupensis quadrasi* based on the *cox1* gene that can be applied in water samples collected from selected schistosomiasis-endemic areas in the Philippines using qPCR based on the TaqMan System.

## Materials and methods

### Study design, malacological survey and field water sampling

**Fig 1:**
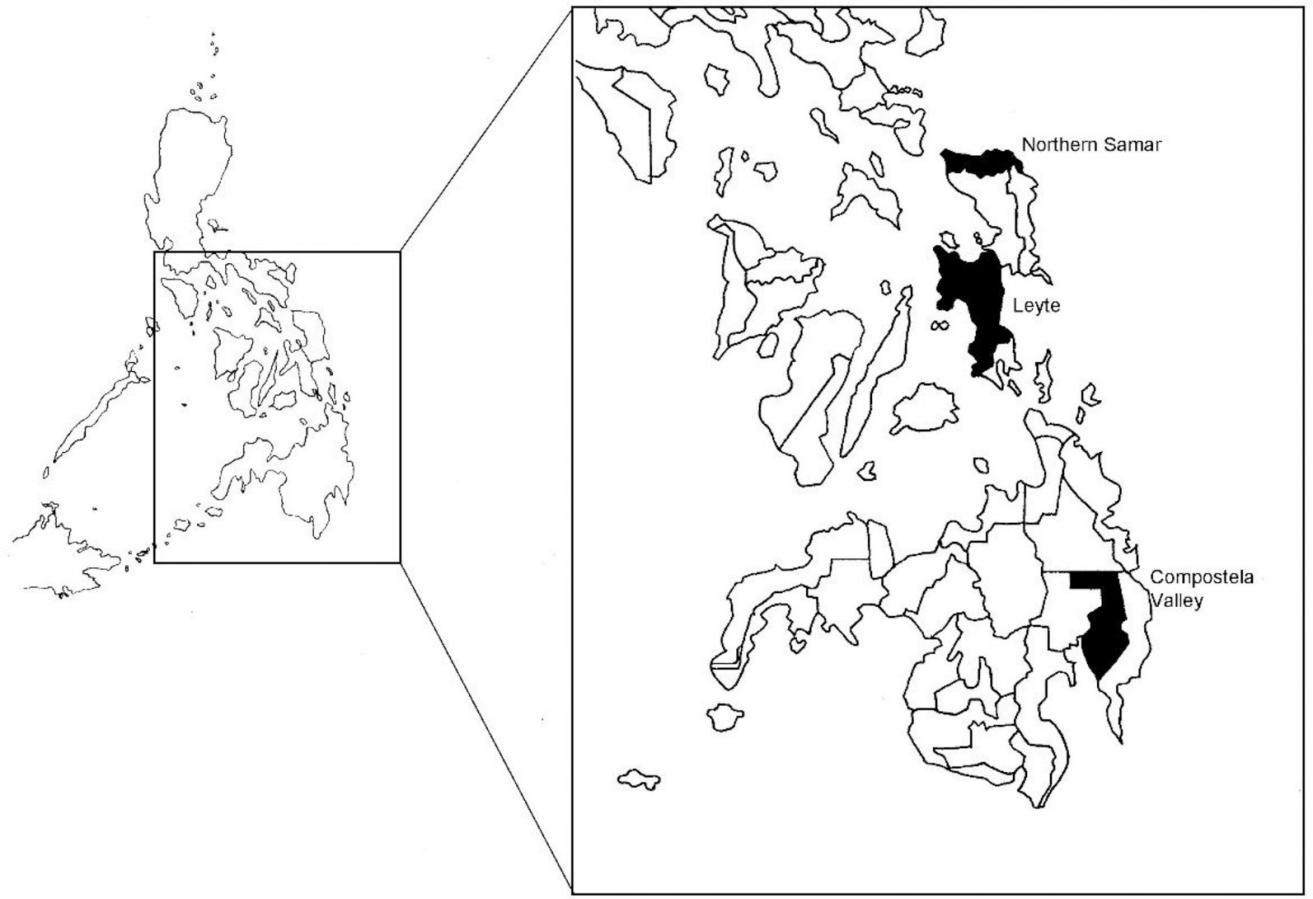
Two schistosomiasis-endemic provinces in the Visayas namely Northern Samar and Leyte and Compostela Valley in Mindanao were surveyed for field water sampling.

Convenient sampling for water collection and malacological survey through manual search of *O*. *hupensis quadrasi* snails were applied in this study. Malacological survey and water collection were performed in identified and potential snail sites in the schistosomiasis endemic provinces of Northern Samar, Leyte, and Compostela Valley from May to November 2017 (Table 1; Fig 1). Malacological survey was performed for at least one hour using forceps and reservoir plastic containers in the collection of snails for each site. After collection, snails were first sorted and counted before crushing. In determining snail infection rate from collected snails, each snail was crushed and examined microscopically. For each glass slide, three equidistant aliquots of a droplet of distilled water were prepared. For each droplet of water, one snail was placed using a pair of forceps. Snails were crushed gently just enough to break the shell by placing another clean glass slide on top of the slide containing the snails. Each aliquot containing a crushed snail was examined using a stereomicroscope and tissues were teased using forceps or a dissecting needle to facilitate release of the sporocysts or the characteristic furcocercous bifurcated cercariae of *S*. *japonicum*, which are indicators of an infected snail [24].

**Table 1:** General site description of sites visited for snail and field water sampling in selected endemic municipalities in Leyte, Northern Samar and Compostela Valley.

Province	Municipality	Site	General Site Description
Leyte	Javier	PINONeg	Uncemented Irrigation Canal
		PINOPos	Swamp, Waterlogged
	Palo	CBRNNeg	Uncemented Irrigation Canal
		CBRNPos	Natural Pool
	Tanauan	GDNSPos	Swamp, Waterlogged
	Pastrana	SOCSNeg	Creek, Flowing
		SOCSPos	Swamp, Waterlogged
	Sta. Fe	CSLNNeg	Swamp, Waterlogged
		CSLNPos	Swamp, Waterlogged
	Julita	DITANeg	Cemented Irrigation Canal, Flowing
		DITAPos	Creek, Flowing
Northern Samar	Catarman	WAS	Creek, Stagnant
		MGS	Creek, Stagnant
		LIBW	Swamp, Waterlogged
		LIBBH	Creek, Flowing
		OLR	Creek, Flowing
Compostela Valley	Maragusan	TIG	Ricefield, Stagnant
		MAP	Creek, Flowing
		NEP	Creek, Flowing

For the water collection, a plastic bottle was used to collect approximately 500 mL of surface water from each site, usually along the edge of the freshwater bodies (e.g. riverbanks and small river tributaries) and within snail sites such as swampy areas with water depth low enough to visit wearing knee-high rubber boots. Gloves were worn by the collectors to prevent exposure to freshwater. A plastic dipper was used to scoop surface water for each site and was washed thoroughly with detergent and distilled water after every sampling. The water samples were immediately stored in a cold container full of ice cubes (approximately 10°C) and were transported to the Rural Health Units for filtration. The water samples were then separately filtered through a circular filter paper (GF/F, 0.7μm; Whatman, Maidstone, UK) using a portable water pump (Nalgene, Sigma-Aldrich, Merck, Germany), which was washed thoroughly after every filtration of samples with detergent and distilled water to prevent cross contamination. After filtration, filter papers were fixed in 70% ethanol [20, 21]. The ethanol-fixed filter papers were packed individually in aluminum foil, labeled and stored at -20°C until further processing.

### Water samples from experimental aquaria

To test whether the designed primers could amplify eDNA in water samples, laboratory set-ups were done using *O*. *hupensis quadrasi* snails collected from the field in a snail site in Guindag-an, Tanauan, Leyte, which was known previously to have very low snail infection rate based on the monitoring data of the Medical Zoology Laboratory of the Schistosomiasis Research and Training Center (SRTC) in Palo, Leyte. Aquaria were prepared using opaque polypropylene tanks (dimensions: length = 20 cm; width = 18.3 cm; height = 10.3 cm). Each tank was filled with 1.5 liters (L) of distilled water and was aerated for 9 hours per day. Water volume was monitored and maintained daily. A total of 12 aquaria were divided into three experimental groups (A, B, C) containing 25, 50, and 75 snails and a negative control group (Neg) that had no snails in triplicates. After four days, the water from each aquarium was filtered in the same way as described previously.

### eDNA extraction

eDNA extraction was performed using DNeasy® Blood & Tissue Kit (Qiagen, Hilden, Germany) as described previously with some modifications [25]. Briefly, each filter paper was transferred and folded carefully using a pair of sterile plastic tweezers and inserted into a labeled salivette tube (Sarstedt, Nümbrecht, Germany). For each tube, 400 μL of Buffer AL and 40 μL Proteinase K was added followed by incubation at 56°C for 30 mins. After incubation, all tubes were centrifuged at 2,500 x *g* for seven minutes. A rinse step using 220 μL of TE buffer was carried out onto the filter papers. After 1 min of incubation at room temperature, the samples were then centrifuged at the same conditions, and 440μL of absolute ethanol was added to the filtrated solution. The binding step was done through standard column extraction by transferring 650 μL of the solution to a DNeasy® column followed by 1 min at 6000 x *g* centrifugation. The binding step was repeated on the entire solution passed through the spin column. Washing and elution followed according to the manufacturer’s protocol. After elution, eDNA extracts were stored at -20°C until further use.

### Primer and probe design

For *S*. *japonicum* primer development, mitochondrial *cox1* sequences of 22 *Schistosoma* species were obtained from NCBI GenBank and a primer set and probe were designed for species-specific eDNA detection of *S*. *japonicum* using Primer Express Software 3.0 (Applied Biosystems, Foster City, CA, USA) with default settings. The forward primer Sj_COI_F (5’-TTTGATAACTAATCACGGTATAGCAA-3’) and the reverse primer Sj_COI_R (5’-CGAGGCAAAGCTAAATCACTC-3’) were designed to amplify approximately 119 bp of the *cox1* gene, which can be visualized using the TaqMan custom probe *S*. *japonicum* (5’-FAM-TTTTGGTAAATATCTTCTTCCG-MGB-NFQ-3’).

For *O*. *hupensis quadrasi* primers were manually designed based on the aligned sequences of *cox1* gene of *O*. *hupensis quadrasi*, other *Oncomelania* subspecies, and related taxa from other gastropod species from the families Planorbidae, Ampullariidae, Neritidae, Achatinidae, and Thiaridae retrieved from Genbank database, done using Clustal W [26] in BioEdit Sequence Alignment Editor 7.0.9.0 [27]. The designed primers sequences were designed to amplify a 187bp of *cox1*, reported by a TaqMan custom probe as follows: forward primer OhqCOX1_22-41aF (5’-GCATGTGAGCGGGGCTAGTA-3’), the reverse primer OhqCOX1_189-209aR (5’- AAGCGGAACCAATCAGTTGCC-3’), and the TaqMan custom probe OhqCOX1_67-86P (5’-FAM-GTGCAGAGTTAGGTCAGTCCT-MGB-NFQ-3’).

### Controls

For the experiments in this study we used four type of samples as controls; (1) PCR positive, (2) non-target DNA control, (3) PCR negative (blank), (4) blank control for the aquarium set-up, (5) PCR negative (water eDNA). All the samples were run in triplicates in different batches or plates to prevent cross contamination.

PCR positive controls are controls with known target DNA of the organism being detected, such as *O*. *hupensis quadrasi* and *S*. *japonicum*. The non-target DNA control are genomic DNA from naturally endemic species in taxa near to our targets. We used as non-target controls *Fasciola* spp., *Melanoides* spp., *Pomacea canaliculata* and *Lymnaea* spp. PCR Negative control is a reaction mixture without any DNA to determine potential contamination during the preparation of PCR reaction mixture. For the laboratory aquaria samples, eDNA from tanks exposed to the same condition but with no snails were used. No specific signal is expected from (2), (3) (4) and (5).

### Ethics

All the samples used in this study (water and snails) were collected in public areas, with no specific permission required or private rice fields with the consent of the owners. None of the parasites and snail’s species used in this study are endangered or protected species.

### Specificity test via endpoint polymerase chain reaction (PCR)

To test for specificities of the primer sets, conventional endpoint PCR (PCR) was performed using genomic DNA (gDNA) of target organisms (*S*. *japonicum* and *O*. *hupensis quadrasi*), non-target snails (*Lymnaea* spp., *Pomacea canaliculata*, and *Melanoides* spp.) and the trematode *Fasciola* spp. that could co-exist in the same environments. The reactions were carried out in a 25 microliter (μL) final volume containing 2.5 μL 1X PCR Buffer, 0.1875 millimolar (mM) dNTP mix, 2 μL 2 mM MgCl_2_, 1.0 μL 0.4 micromolar (μmol) each of forward and reverse primers, 0.125 μL 0.625 units (U) of *Taq* Polymerase (Takara-Clontech, Kusatsu, Shiga, Japan), 2.0 μL (1.1–79.4 ng/μL) genomic DNA (gDNA), and 14.5 μL nuclease-free PCR grade water. Cycling conditions for the PCR consisted of a 2 min denaturation step at 94°C, followed by 35 cycles of denaturation at 98°C for 10 secs, annealing at 60°C (*O*. *hupensis quadrasi*)/ 55°C (*S*. *japonicum*) for 30 secs, and extension at 68°C for 30 secs, and final extension at 72°C for 7 mins. PCR products were detected in a 2% agarose gel stained with 1% ethidium bromide (EtBr) using TBE (Tris, Boric Acid, EDTA) as the electrode buffer and were run in 100 volts for 30 mins. Distinct bands were purified using a commercial gel purification kit (QIAquick® Gel Extraction Kit, QIAGEN, Hilden, Germany) following the manufacturer’s protocol. Purified PCR products were sent to Macrogen Inc. South Korea for single pass sequencing. Sequences were assembled using Staden package [28] and BLASTn® search (https://blast.ncbi.nlm.nih.gov) were performed for generated consensus sequences for confirmation of sequence identity [29].

### qPCR assay of laboratory and field water samples

The presence of *S*. *japonicum* and *O*. *hupensis quadrasi* DNA in the samples were analyzed using the designed TaqMan probe-based real-time PCR. All assays were performed using Thermal Cycler Dice® Real Time System II (TP 900) in a 25 μL reaction volume consisting of 1X Premix Ex Taq (Probe qPCR), 0.2 μM each of the forward and reverse primers, 0.4 μM of TaqMan probe (Eurofins Genomics, Keihinjima,Ota-ku, Tokyo, Japan), and 2 μL (1.1–79.4 ng/μL) of eDNA sample. The PCR conditions were initial denaturation at 95°C for 5 secs followed by 50 cycles of two-step PCR at 95°C for 5 secs and 60°C for 30 secs. All samples were tested in triplicates and included the negative controls and a positive (gDNA of *S*. *japonicum* and *O*. *hupensis quadrasi*) control. Amplification in at least one replication was considered positive [21].

### Comparison of qPCR method and malacological survey

For analysis of field water samples, the sites were further classified based on the results of snail crushing and malacological survey from the qPCR results of water collected for the detection of S. *japonicum* and *O*. *hupensis quadrasi*.

## Results

### PCR optimization and specificity

**Fig 2:**
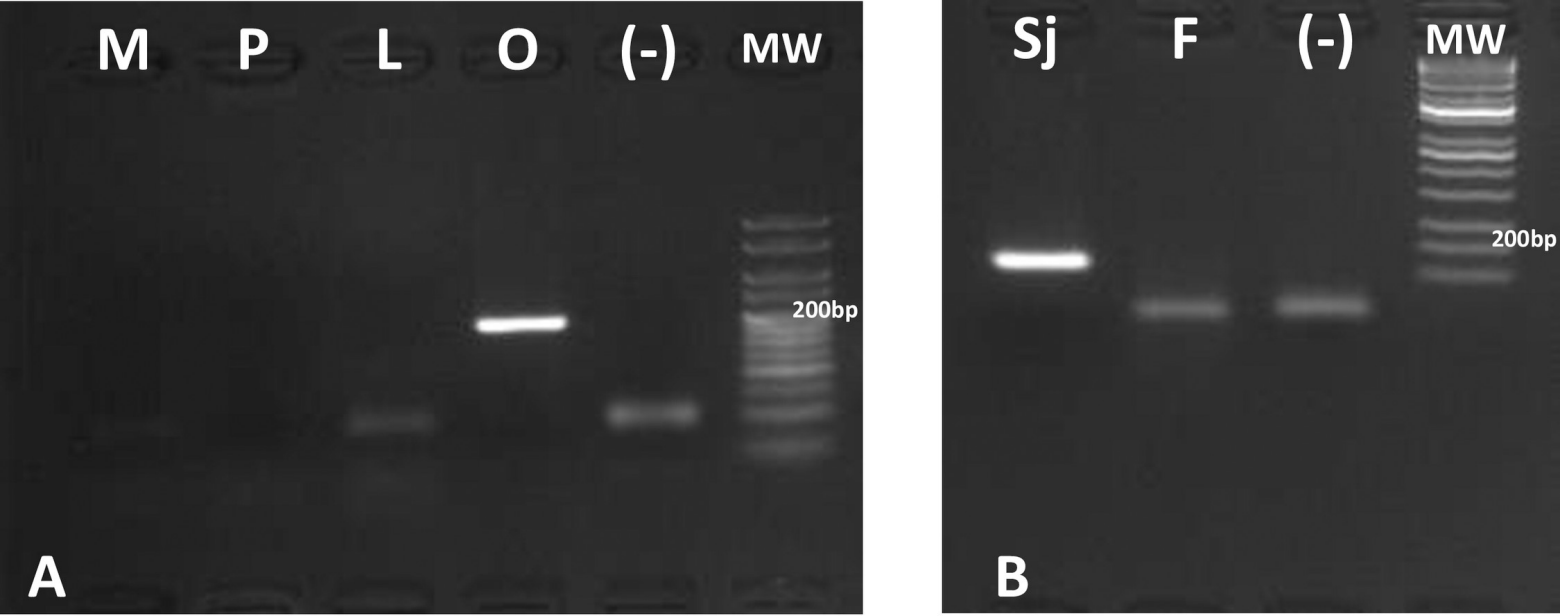
Specificity test by PCR using designed *O*. *hupensis quadrasi* (A) and *S*. *japonicum* (B) primers. On the left (A) the PCR templates were the gDNA of *O*. *hupensis quadrasi* (lane O) and the non-target gDNA of *Melanoides* spp. (lane M), *Pomacea canaliculata* (lane P) and *Lymnaea* spp. (lane L). On the right (B) the PCR using *S*. *japonicum* primers were done with templates of *S*. *japonicum* gDNA (lane Sj), and the non-target gDNA of *Fasciola* spp. (F). The PCR blank and the molecular weight ladder are indicated as (-) and MW in A and B.

For both *S*. *japonicum* and *O*. *hupensis quadrasi* primers, BLAST® search was performed to determine sequence specificity. For *S*. *japonicum* primers, top hits for forward and reverse primer BLAST® are *S*. *japonicum cox1* gene and no other trematode species was shown in the search. For *O*. *hupensis quadrasi*, top hit results are *O*. *hupensis quadrasi* and *O*. *hupensis hupensis* for both forward and reverse primers. Results of primer specificity for both *S*. *japonicum* and *O*. *hupensis quadrasi* yielded negative results for non-target specimen using endpoint PCR (Fig 2). Further confirmation on PCR assay from target gDNA samples via single pass sequencing of PCR products validated the target specificity of the primers (data not shown).

### qPCR analysis of laboratory and field water samples

Based on qPCR results, all aquaria except the negative set-ups were positive to *O*. *hupensis quadrasi* DNA, while 7 out of the 9 aquaria had detectable *S*. *japonicum* DNA (Table 2).

**Table 2:** Taqman real-time qPCR results of water from experimental ecotopes with different numbers of snails utilizing *S*. *japonicum* and *O*. *hupensis quadrasi* specific primers in a TaqMan system tested in triplicates.

Groups	Number of Snails per aquarium	TaqMan qPCR	
		*S*. *japonicum*	*O*. *hupensis quadrasi*
1A	25	+	+
1B		+	+
1C		-	+
2A	50	-	+
2B		+	+
2C		+	+
3A	75	+	+
3B		+	+
3C		+	+
Neg	-	-	-

Regarding the field water samples, several sites tested positive for DNA of *S*. *japonicum* (9/19), *O*. *hupensis quadrasi* (9/19) or both (5/19) (Table 3). *O*. *hupensis quadrasi* eDNA was detected in the field for both snail-positive and negative sites based from the malacological surveys conducted (Table 3). Sites that were found to be positive for *O*. *hupensis quadrasi* through malacological survey are usually established and previously known snail infested areas while sites that are negative for the snails could actually harbor snail colonies even if no snails were found at the time of survey. These sites manifested conditions suitable for the snails such as being waterlogged and well-shaded. In sites where no snails were found, three sites were recorded positive for *O*. *hupensis quadrasi* eDNA (Table 3).

**Table 3:** Summary results of intensive malacological survey per site, determination of infection rate and TaqMan qPCR detection of *S*. *japonicum* and *O*. *hupensis quadrasi* eDNA from field water samples.

Site	Malacological Survey	% Infection Rate	TaqMan qPCR		Agreement of Conventional Survey vs. qPCR
		Number of Snails (Infected/Examined)	*S*. *japonicum*	*O*. *hupensis quadrasi*	
PINOPos	+	2.08	+	+	*S*. *japonicum* (80%) (4 sites positive in qPCR / 5 sites positive to *S*. *japonicum* cercariae determined via snail crushing) x 100 *ND sites not included
		(17/819)			
GDNSPos	+	0.43	+	+	
		(3/697)			
CBRNPos	+	1.41	+	+	
		(6/427)			
SOCSPos	+	0.24	+	+	
		(2/819)			
CSLNPos	+	0.20	-	-	*O*. *hupensis quadrasi* (85.71%) (6 sites positive in qPCR/7 sites positive to *O*. *hupensis quadrasi* snails via malacological survey) x 100
		(1/505)			
DITAPos	+	**ND	+	+	
TIG	+	**ND	+	+	
PINONeg	-	-	+	-	
CBRNNeg	-	-	-	-	*O*. *hupensis quadrasi* (25%) (3 sites positive in qPCR / 12 sites negative to *O*. *hupensis quadrasi* snails determined via malacological survey) x 100
SOCSNeg	-	-	-	-	
DITANeg	-	-	-	-	
CSLNNeg	-	-	-	+	
WAS	-	-	-	+	
MGS	-	-	+	-	
LIBW	-	-	-	-	
LIBBH	-	-	-	-	
OLR	-	-	-	+	
MAP	-	-	+	-	
NEP	-	-	-	-	

*A minimum of 1 positive result over 3 repeated runs per each sample is considered positive.

**Not Determined due to dead snails

Detection rates of the qPCR assays were compared to malacological survey and presence of *S*. *japonicum* determined by snail crushing (Table 3). Five sites with snails found to be infected with *S*. *japonicum* cercariae confirmed through snail crushing were included in calculating percent detection rate for the presence of *S*. *japonicum*. From these five field water samples, three were positive for *S*. *japonicum* by qPCR. Six out of seven filtered water samples previously detected to be positive to *O*. *hupensis quadrasi* by malacological survey were positive by qPCR. Moreover, three from 12 water samples from sites previously noted as negative to *O*. *hupensis quadrasi* through malacological survey were positive by qPCR. Since no snails could be found in PINONeg, CBRNNeg, SOCSNeg, DITANeg, CSLNNeg, WAS, MGS, LIBW, LIBBH, OLR, MAP and NEP, infection rate data was not generated for these sites. Moreover, certain sites were noted positive to *S*. *japonicum* eDNA such as PINONeg, MGS, and MAP (Table 3).

## Discussion

Monitoring and elimination of schistosomiasis japonica depends on rapid, accurate and comprehensive analysis of data on *S*. *japonicum* and *O*. *hupensis quadrasi* in the environment. Data on snail habitats, snail population densities, and snail infection rates are most relevant in planning intervention measures. The dependence of schistosome parasites and their snails host on water has provided an avenue to detect their presence in water through eDNA, as shown by previous studies, especially in the Philippines were this method has not been applied previously.

The use of barcoding markers such as *cox1* enables accurate identification of *S*. *japonicum* and *O*. *hupensis quadrasi* through non-invasive means of surveying their potential presence and distribution in an ecosystem [19,30]. Species specific DNA detection using environmental samples is an emerging technique for biodiversity estimation by analyzing environmental samples which may contain biomass of various target organisms of interest [31].

The origin of environmental DNA is diverse. It is found in cells or free DNA and could be derived from desquamation cells, reproduction related cells, degraded cells, etc. Primers that target short fragments of DNA are known to optimally detect eDNA from environmental samples since eDNA are expected to be fragmented due to exposure to various environmental stressors [32]. Though eDNA is influenced by physiological, chemical, physical and biological factors in the environment for species detection, modified adaptive sampling techniques to optimize efficient capture of eDNA can be done to increase its utility [33]. eDNA decay, or the persistence of DNA in various matrices, should also be considered to efficiently delineate signals from live organisms and decaying carcasses for a more accurate real-time data of distribution of target species [34]. In the case of eDNA detection of snails and *S*. *japonicum* in this study, it should follow the same characteristics of origin and decay in the environment of the other organisms. Therefore, due to the fast degradation of eDNA in natural conditions, the encounter of this direct evidence means the organisms were present in the moment of the sample collection. Nevertheless, this new technology offers an immense applicability and utility in parasite monitoring and detection in the natural environment.

Compared to other *O*. *hupensis* subspecies, *O*. *hupensis quadrasi* is the most amphibious and can be seen on soil, buried in mud, clinging to leaf and stalks of surrounding vegetation or even submerged underwater [5,35–37]. The amphibious property of the snail and its frequent interaction with freshwater makes it susceptible to dispersal from intense water flow most especially during flooding [38]. Newly formed waterlogged bodies in endemic areas may serve as new snail breeding sites for dispersed populations of snails after flooding [39]. Snails can also be transported through migratory birds or through farm animals like water buffalos where snails may attach to the skin or hooves when these animals wallow in the mud or water [40]. Emergence of new snail sites can contribute to spatial extension of transmission sites, hence the necessity of snail surveillance and mapping out these new sites to be added to the established list of snail sites [35]. Control, prevention and elimination of the disease call for continuous monitoring of previously identified snail sites and searching for new potential snail breeding sites. This could mean covering huge tracts of grassy marshlands, swamps, lagoons, ponds, irrigation canals, streams and small tributaries of rivers. The manpower involved in this immense task and the risk involved to schistosome infection as well as accidents in accessing these areas cannot be overestimated and definitely have an effect on the quality of data generated from malacological surveys if these challenges are not well addressed [3,7]. Though eDNA assays still requires going to these sites, water collection is still comparatively easier to perform as these can be collected randomly even without *a priori* knowledge in the exact locations of snail colonies within the freshwater water body [23].

In this study, a 119 bp *S*. *japonicum* and 187 bp *O*. *hupensis quadrasi cox1* fragments were utilized as targets for eDNA detection from these organisms. qPCR using TaqMan system is a useful tool for rapid and sensitive detection of target DNA from various sources such as freshwater samples, which may contain high concentrations of non-target DNA and other inhibitors. This study further demonstrated the utility of qPCR detection by providing the first set of *cox1* markers for *S*. *japonicum* and *O*. *hupensis quadrasi*. Successful detection of *O*. *hupensis quadrasi* from water obtained from aquaria with definite number of snails submerged under controlled conditions was also demonstrated. In one part of this study, snails were collected from a site which was previously recorded with very low to zero infection rates. qPCR, however, detected *S*. *japonicum* eDNA in the water of the tanks where these snails were kept. It is possible that these snails could have shed cercariae during the four days that they were kept in the tank. Further confirmation of snails in aquaria that tested positive for *S*. *japonicum* could have been done if snails were crushed after to test presence of sporocysts or cercariae, but this was not performed in the study.

In addition, field water samples collected from sampling points which were negative for *O*. *hupensis quadrasi* based on malacological survey turned out to be positive for eDNA as shown by qPCR results (Table 3). Such observation can possibly be attributed to the mobility of eDNA, allowing it to be translocated based on water flow [36]. It is possible that *S*. *japonicum* and *O*. *hupensis quadrasi* eDNA detected from these sites originated from a source hydrologically connected to the sampled area, or simply we did not find the snails. This is very important in schistosomiasis control especially in determining the spatial range within which cercariae may be carried or transported to other areas where they can infect unsuspecting hosts. It would be logical to establish a specific radius from the snail site where infected snails were found and assign this as potential transmission site where water contact can pose infection risk [1,2,6]. It is also equally possible that detection of *S*. *japonicum* in sites negative through malacological surveys may have been detected from miracidia, yet the qPCR performed could not differentiate the specific life stages from *S*. *japonicum* since the method is DNA-based. Moreover, comparison of *S*. *japonicum* eDNA detection using qPCR to snail crushing were shown. Four field water samples collected from snail sites out of five sites confirmed through snail crushing were detected positive to *S*. *japonicum* eDNA, indicating a qPCR sensitivity of 80.0% (Table 3). In a recent study of Sato and co-workers (2018) on *S*. *mansoni* detection in Madagascar, water samples that were *S*. *mansoni* eDNA-positive were also observed to have *B*. *pfeifferi* snails. This observation reinforces the link between the parasite and its snail intermediate host, which can also be applied to *S*. *japonicum* and *O*. *hupensis quadrasi*.

The strategy of eDNA detection for schistosomiasis japonica intermediate host may seem to be different of other intermediate hosts of schistosomiasis such as for *S*. *mansoni*, *S*. *intercalatum* and *S*. *haematobium*, which are purely aquatic. The amphibious behaviour of *O*. *hupensis quadrasi* has an important epidemiological value due to the difficulty to detect snails in dry sites that were initially waterlogged. Some sites may appear clear of snails at malacological inspection, but these dry sites after rehydration through the coming of rainy season or through flooding, allows snail colonies to be reestablished. Our system is directed to detect snails eDNA in water samples so it has limited use in dry situations or areas with dry and wet places. Recently, a study conducted by Calata and co-workers (2019) demonstrated the utility of soil as a material to detect *O*. *hupensis quadrasi* eDNA in Gonzaga, Cagayan Valley in Northern Philippines [41]. This enables the detection of the snail even in dry areas most especially that this region in the Philippines experiences drought and relatively higher temperatures during summer than the rest of the country.

Results of this study provide evidence that eDNA analysis can complement conventional malacological methods used and, at the same time, provide basis for inferring the possible extent of spread of schistosome miracidia, cercariae or the snails themselves [36,37]. For instance, eDNA may be detected from a site where there are no actual *O*. *hupensis quadrasi* snails. It could be inferred that the eDNA might be coming from an upstream source of snail colony shedding eDNA [41]. Since successful transmission of *S*. *japonicum* involves convergence of certain elements of the life cycle such as miracidia infecting the snails and their eventual shedding as cercariae in the surrounding water, systematic water sampling in snail sites may increase chances of detecting eDNA either from *S*. *japonicum* or *O*. *hupensis quadrasi*. Absence of snails after surveying a site should therefore not be used as the only indicator of the absence of snails since successful recovery of snails is not always assured in manual search. This was proven in this study by the detection of eDNA in water samples collected from snail areas which have been declared snail-free based on malacological survey. It is also suggested to determine eDNA shedding and decay rate of both *S*. *japonicum* and *O*. *hupensis quadrasi* to eventually quantify and translate qPCR cycle threshold (ct) values to number of individuals could be an important eco-epidemiological information for schistosomiasis control. Studies have also reported the utility of eDNA in estimating the size and number of target organism in aquatic ecosystems such as for economically-important marine fish species [35]. For schistosomiasis japonica, this will give a strong predictive tool for estimating snail density in sampled snail habitats. Aside from eDNA shedding, eDNA decay should be determined to estimate real-time distributions of the snail intermediate host and the range or distance that either miracidia or cercariae of *S*. *japonicum* can reach from its source in freshwater bodies. These real-time information are helpful in creating intervention plans ranging from formulating risk and hazard maps and in prioritizing human and animal morbidity control in areas with high detection rates of the parasites/intermediate hosts in the environment.

The presence of eDNA of either the parasite, the snail, or both in a water body can provide information about the risk of exposure that contact with such water body can cause. Despite it is not possible to determine specifically the existence of cercaria in the water, the use of eDNA detection enable us to determine the presence of the parasite and its intermediate host as well. Further, it can also validate the near-elimination status from schistosomiasis japonica of certain areas such as Talibon and Trinidad in Bohol province and Davao City in Mindanao. The method developed could therefore be utilized in snail and parasite surveillance which is certainly critical in determining progress and success of intervention measures implemented in endemic areas.

## Supporting information

S1 Table Dataset containing samples description, Ct results of qPCR and the coordinates of each collection site. (DOCX)
