# Supplementary material for: Detection of Schistosoma japonicum and Oncomelania hupensis quadrasi environmental DNA and its potential utility to schistosomiasis japonica surveillance in the Philippines
Source: PLoS One. 2019 Nov 20;14(11):e0224617. doi: 10.1371/journal.pone.0224617 (PMC6867693; doi:10.1371/journal.pone.0224617)
Supplement: S1 Table — (DOCX) [file pone.0224617.s001.docx]

S1 Table

| **Sample Description** | | | | | ***O. h. quadrasi*** | ***S. japonicum*** | **Coordinates** | | |  |
| --- | --- | --- | --- | --- | --- | --- | --- | --- | --- | --- |
| Target Name | Sample ID | Sample Name | Nucleic Acid Conc. (ng/uL) | Filter | Ct | Ct | N | E | |  |
| CO1 | 1 | PINONeg1 | 6.4 | FAM | -- | 39.2 | 10° 47.911' | 124° 57.029' | |  |
| CO1 | 9 | CBRNPos4 | 7.4 | FAM | -- | -- | 11° 06.377' | 124° 57.074' | |  |
| CO1 | 17 | SOCSNeg2 | 1.9 | FAM | -- | -- | 11° 08.731' | 124° 53.291' | |  |
| CO1 | 25 | DITAPos2 | 5.1 | FAM | -- | -- | 10° 58.487' | 124° 56.987' | |  |
| CO1 | 33 | GDNSPos2 | 10.2 | FAM | -- | -- | 11° 06.079' | 124° 58.192' | |  |
| CO1 | 41 | WAS1B | 19.9 | FAM | -- | -- | 12° 27.755' | 124° 38.522' | |  |
| CO1 | 49 | OLRD | 4.4 | FAM | -- | -- | 12° 30.477' | 124° 35.490' | |  |
| CO1 | 57 | MGS2 | 6.1 | FAM | -- | -- | 12° 29.077' | 124° 35.490' | |  |
| CO1 | 65 | 25Saq2 | 27.7 | FAM | 28.88 | -- | Laboratory Set-up | | |  |
| CO1 | 73 | rtPosOL3 | 10 | FAM | 22.68 | 33.56 | Real Time PCR Positive Control | | |  |
| CO1 | 2 | PINONeg2 | 5.6 | FAM | -- | -- | 10° 47.911' | 124° 57.029' | |  |
| CO1 | 10 | CBRNPos5 | 1.5 | FAM | -- | -- | 11° 06.377' | 124° 57.074' | |  |
| CO1 | 18 | SOCSNeg3 | 1.8 | FAM | -- | -- | 11° 08.731' | 124° 53.291' | |  |
| CO1 | 26 | DITAPos3 | 2.4 | FAM | -- | -- | 10° 58.487' | 124° 56.987' | |  |
| CO1 | 34 | GDNSPos3 | 5.4 | FAM | -- | -- | 11° 06.079' | 124° 58.192' | |  |
| CO1 | 42 | WAS1C | 1.2 | FAM | -- | -- | 12° 27.755' | 124° 38.522' | |  |
| CO1 | 50 | NEPA | 4.9 | FAM | -- | -- | 07° 16.367' | 126° 06.743' | |  |
| CO1 | 58 | MGS3 | 2.4 | FAM | -- | -- | 12° 29.077' | 124° 35.490' | |  |
| CO1 | 66 | 25Saq3 | 39.1 | FAM | 28.43 | -- | Laboratory Set-up | | |  |
| CO1 | 74 | rtNeg1 | - | FAM | -- | -- | Real Time PCR Negative Control | | |  |
| CO1 | 3 | PINONeg3 | 4.7 | FAM | -- | -- | 10° 47.911' | 124° 57.029' | |  |
| CO1 | 11 | CBRNPos2 | 9.5 to 14.5 | FAM | -- | 34.47 | 11° 06.377' | 124° 57.074' | |  |
| CO1 | 19 | SOCSNeg4 | 3.6 | FAM | -- | -- | 11° 08.731' | 124° 53.291' | |  |
| CO1 | 27 | DITAPos4 | 5 | FAM | -- | -- | 10° 58.487' | 124° 56.987' | |  |
| CO1 | 35 | GDNSPos4 | 11.3 | FAM | 36.63 | -- | 11° 06.079' | 124° 58.192' | |  |
| CO1 | 43 | WAS1D | 1.9 | FAM | -- | -- | 12° 27.755' | 124° 38.522' | |  |
| CO1 | 51 | NEPB | 1.1 | FAM | -- | -- | 07° 16.367' | 126° 06.743' | |  |
| CO1 | 59 | LIB1W | 78.2 | FAM | -- | -- | 12° 29.922' | 124° 36.569' | |  |
| CO1 | 67 | 50Saq1 | 67.8 | FAM | 29.82 | -- | Laboratory Set-up | | |  |
| CO1 | 75 | rtNeg2 | 10 | FAM | -- |  | Real Time PCR Negative Control | | |  |
| CO1 | 4 | PINONeg4 | 2.6 | FAM | -- | -- | 10° 47.911' | 124° 57.029' | |  |
| CO1 | 12 | CBRNPos3 | 2.4 | FAM | -- | -- | 11° 06.377' | 124° 57.074' | |  |
| CO1 | 20 | SOCSNeg5 | 3 | FAM | -- | -- | 11° 08.731' | 124° 53.291' | |  |
| CO1 | 28 | DITANeg1 | 13.7 | FAM | -- | -- | 10° 58.625' | 124° 56.797' | |  |
| CO1 | 36 | CSLNPos1 | 7.3 | FAM | -- | -- | 11° 09.752' | 124° 53.928' | |  |
| CO1 | 44 | WAS2A | 1.5 | FAM | -- | -- | 12° 27.755' | 124° 38.522' | |  |
| CO1 | 52 | TIGA | 2.8 | FAM | -- | 36.98 | 07° 15.690' | 126° 07.831' | |  |
| CO1 | 60 | LIB2W | 79.4 | FAM | -- | -- | 12° 29.922' | 124° 36.569' | |  |
| CO1 | 68 | 50Saq2 | 47.9 | FAM | 29.18 | 34.19 | Laboratory Set-up | | |  |
| CO1 | 76 | sJPOS1 | 10 | FAM | 18.82 |  | Real Time PCR Positive Control | | |  |
| CO1 | 5 | PINOPos1 | 3.3 | FAM | -- | -- | 10° 47.910' | 124° 57.031' | |  |
| CO1 | 13 | CSLNNeg1 | 8.3 | FAM | -- | -- | 11° 09.854' | 124° 53.887' | |  |
| CO1 | 21 | SOCSPos1 | 2.9 | FAM | -- | -- | 11° 08.739' | 124° 53.291' | |  |
| CO1 | 29 | DITANeg2 | 14.3 | FAM | -- | -- | 10° 58.625' | 124° 56.797' | |  |
| CO1 | 37 | CSLNPos2 | 6 | FAM | -- | -- | 11° 09.752' | 124° 53.928' | |  |
| CO1 | 45 | WAS2B | 4.4 | FAM | -- | -- | 12° 27.755' | 124° 38.522' | |  |
| CO1 | 53 | TIGB | 3.3 | FAM | 38.06 | 34.85 | 07° 15.690' | 126° 07.831' | |  |
| CO1 | 61 | LIB3BH | 5.9 | FAM | -- | -- | 12° 29.356' | 124° 36.223' | |  |
| CO1 | 69 | 50Saq3 | 28.5 | FAM | 32.39 | -- | Laboratory Set-up | | |  |
| CO1 | 77 | sjPOS2 | 10 | FAM | 19.96 |  | Real Time PCR Positive Control | | | |
| CO1 | 6 | PINOPos2 | 5.9 | FAM | -- | -- | 10° 47.910' | | 124° 57.031' | |
| CO1 | 14 | CSLNNeg2 | 25.2 | FAM | -- | -- | 11° 09.854' | | 124° 53.887' | |
| CO1 | 22 | SOCSPos2 | 3.5 | FAM | -- | -- | 11° 08.739' | | 124° 53.291' | |
| CO1 | 30 | DITANeg3 | 10 | FAM | -- | -- | 10° 58.625' | | 124° 56.797' | |
| CO1 | 38 | CBRNNeg1 | 7.8 | FAM | -- | -- | 11° 06.408' | | 124° 57.035' | |
| CO1 | 46 | OLRA | 6.1 | FAM | -- | -- | 12° 30.477' | | 124° 35.490' | |
| CO1 | 54 | MAPA | 2.1 | FAM | -- | -- | 07° 18.233' | | 126° 08.900' | |
| CO1 | 62 | LIB4BH | 4.6 | FAM | -- | -- | 12° 29.356' | | 124° 36.223' | |
| CO1 | 70 | 75Saq1 | 13.4 to 13.6 | FAM | 32.43 | 31.2 | Laboratory Set-up | | | |
| CO1 | 7 | PINOPos3 | 5.2 | FAM | -- | -- | 10° 47.910' | | 124° 57.031' | |
| CO1 | 15 | CSLNNeg3 | 7.7 | FAM | -- | -- | 11° 09.854' | | 124° 53.887' | |
| CO1 | 23 | SOCSPos3 | 2.9 | FAM | -- | -- | 11° 08.739' | | 124° 53.291' | |
| CO1 | 31 | DITANeg4 | 16.4 | FAM | -- | -- | 10° 58.625' | | 124° 56.797' | |
| CO1 | 39 | CBRNNeg2 | 7 | FAM | -- | -- | 11° 06.408' | | 124° 57.035' | |
| CO1 | 47 | OLRB | 5.8 | FAM | 30.47 | -- | 12° 30.477' | | 124° 35.490' | |
| CO1 | 55 | MAPB | 3.6 | FAM | -- | -- | 07° 18.233' | | 126° 08.900' | |
| CO1 | 63 | SaqNeg | 1.9 | FAM | -- | -- | Laboratory Set-up | | | |
| CO1 | 71 | 75Saq2 | 10.1 | FAM | 32.27 | 30.25 | Laboratory Set-up | | | |
| CO1 | 8 | CBRNPos1 | 14.7 | FAM | -- | -- | 11° 06.377' | | 124° 57.074' | |
| CO1 | 16 | SOCSNeg1 | 2.7 | FAM | -- | -- | 11° 08.731' | | 124° 53.291' | |
| CO1 | 24 | DITAPos1 | 6.1 | FAM | -- | -- | 10° 58.487' | | 124° 56.987' | |
| CO1 | 32 | GDNSPos1 | 10.2 | FAM | 37.99 | -- | 11° 06.079' | | 124° 58.192' | |
| CO1 | 40 | WAS1A | 12.5 | FAM | -- | -- | 12° 27.755' | | 124° 38.522' | |
| CO1 | 48 | OLRC | 5 | FAM | -- | -- | 12° 30.477' | | 124° 35.490' | |
| CO1 | 56 | MGS1 | 24.6 | FAM | -- | -- | 12° 29.077' | | 124° 35.490' | |
| CO1 | 64 | 25Saq1 | 38.3 | FAM | 31.05 | -- | Laboratory Set-up | | | |
| CO1 | 72 | 75Saq3 | 30.9 | FAM | 33.11 | 30.97 | Laboratory Set-up | | | |

| Legend: |  |
| --- | --- |
|  | (+) *O. h. quadrasi* eDNA |
|  | (+) *S. japonicum* eDNA |
|  | (+) to both |
